# Mechanistic Insights into Elastin Degradation by Pseudolysin, the Major Virulence Factor of the Opportunistic Pathogen *Pseudomonas aeruginosa*

**DOI:** 10.1038/srep09936

**Published:** 2015-04-23

**Authors:** Jie Yang, Hui-Lin Zhao, Li-Yuan Ran, Chun-Yang Li, Xi-Ying Zhang, Hai-Nan Su, Mei Shi, Bai-Cheng Zhou, Xiu-Lan Chen, Yu-Zhong Zhang

**Affiliations:** 1 State Key Laboratory of Microbial Technology, Shandong University, Jinan 250100, China; 2 Biotechnology Research Center, Shandong University, Jinan 250100, China; 3 Collaborative Innovation Center of Deep Sea Biology, Shandong University, Jinan 250100, China

## Abstract

Pseudolysin is the most abundant protease secreted by *Pseudomonas aeruginosa* and is the major extracellular virulence factor of this opportunistic human pathogen. Pseudolysin destroys human tissues by solubilizing elastin. However, the mechanisms by which pseudolysin binds to and degrades elastin remain elusive. In this study, we investigated the mechanism of action of pseudolysin on elastin binding and degradation by biochemical assay, microscopy and site-directed mutagenesis. Pseudolysin bound to bovine elastin fibers and preferred to attack peptide bonds with hydrophobic residues at the P1 and P1’ positions in the hydrophobic domains of elastin. The time-course degradation processes of both bovine elastin fibers and cross-linked human tropoelastin by pseudolysin were further investigated by microscopy. Altogether, the results indicate that elastin degradation by pseudolysin began with the hydrophobic domains on the fiber surface, followed by the progressive disassembly of macroscopic elastin fibers into primary structural elements. Moreover, our site-directed mutational results indicate that five hydrophobic residues in the S1-S1’ sub-sites played key roles in the binding of pseudolysin to elastin. This study sheds lights on the pathogenesis of *P. aeruginosa* infection.

P*seudomonas aeruginosa* is an opportunistic human pathogen that can cause eye infections, pulmonary infections, bacteremic infections and burn infections. Pseudolysin is the most abundant protease secreted by *P. aeruginosa,* and is considered the predominant extracellular virulence factor of *P. aeruginosa*^1^. Pseudolysin is responsible for the destruction of arterial, elastin lamina in *Pseudomonas* septicemia and corneal damage in keratitis^2^,^3^. Wretlind and Pavlovskis have reviewed the roles of pseudolysin in *P. aeruginosa* infections and have determined that pseudolysin has tissue-damaging, lethal, and cytotoxic effects and interferes with host defense mechanisms when it is injected into specific organs^4^. In general, pseudolysin assists in the colonization and establishment of infection by *P. aeruginosa* in various infections in humans^2^,^5^. Pseudolysin is a well-known bacterial elastase, displaying high specific activity toward elastin, which is probably the main pathway through which it destroys human tissues. Therefore, it is important to determine the elastinolytic mechanism of pseudolysin to further understand the pathogenesis of *P. aeruginosa* infection.

Pseudolysin is a Zn^2+^ metalloprotease of the thermolysin family. It is an endopeptidase active at neutral pH. Its specificity has been determined using various synthetic peptides^6^ and a focused library of N-alpha mercaptoamide-containing dipeptides as inhibitors^7^,^8^, which show that pseudolysin has a preference for aromatic and/or large aliphatic amino acids at the P1’ position and a distinct bias against acidic residues at the P2’ position. The structure of pseudolysin complexed with N-(1-carboxy-3-phenylpropyl)-phenylalanyl-alpha-asparagine (HPI) (PDB id: 1U4G) was reported in 1991^9^. HPI is bound in the S1-S1’ sub-sites of pseudolysin by hydrogen bonding and hydrophobic and weak van der Waal’s interactions^10^. The S1’ sub-site is a deep hydrophobic pocket and is the principal determinant of pseudolysin specificity^9^,^10^,^11^. The S1 sub-site is a partially hydrophilic pocket^10^. Although it has long been known that pseudolysin exhibits high elastinolytic activity, the mechanism of action of pseudolysin on elastin fibers remains unknown.

Elastin fibers are abundant in elastin connective tissues in the human body, such as the skin, lung, aorta and elastin arteries, ligaments and auricular cartilage^12^,^13^,^14^,^15^. Tropoelastin is the fundamental building component of elastin fiber. The sequence of tropoelastin is generally composed of two major types of domains, hydrophobic and hydrophilic, which alternate in the sequence. The hydrophobic domains are rich in nonpolar residues and involved in the alignment of tropoelastin, whereas the hydrophilic domains are mostly composed of Lys and Ala and participate in cross-linking^16^. The process of insoluble elastin formation mainly involves the coacervation of soluble tropoelastin molecules (~15 nm) into micron-sized spherules and lysyl oxidase-mediated cross-linking of the spherules^17^,^18^,^19^. After complex cross-linking, the insoluble elastin fibers are resistant to most proteases and are only sensitive to a limited number of elastases. The major function of elastin fibers is to endow tissues with elastin resilience and recoil. These fibers also regulate the activity of growth factors in elastin tissues and directly regulate cell attachment, thereby mediating cell migration, survival and differentiation^20^. Considering the abundance and significance of elastin fibers in the human body and the high elastinolytic activity of pseudolysin, it is important to explore the structural and mechanical consequences of elastin fiber degradation by pseudolysin.

In this study, using bovine elastin fibers as a model, we report the mechanism of elastin fiber degradation by pseudolysin. Pseudolysin molecules could bond to insoluble bovine elastin fibers and preferred to attack the peptide bonds with hydrophobic residues at the P1 and P1’ positions in the hydrophobic domains of elastin. Using light microscopy (LM) and scanning electron microscopy (SEM), we monitored the degradation process of bovine elastin fibers and cross-linked human tropoelastin by pseudolysin. Moreover, we analyzed the key amino acid residues in pseudolysin responsible for elastin binding via site-directed mutagenesis. Our results reveal the elastinolytic mechanism of pseudolysin, which aids the understanding of the mechanism of *P. aeruginosa* infection.

## Results

### Binding of pseudolysin to insoluble elastin

**Figure 1:**
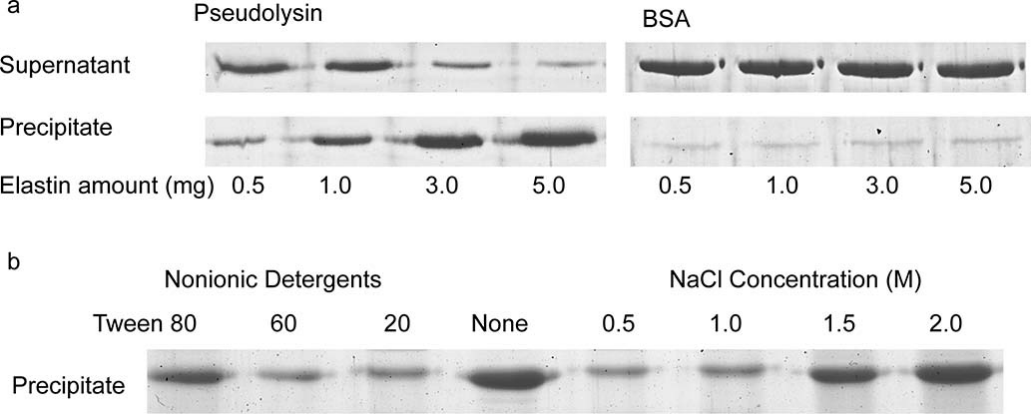
Binding of pseudolysin to insoluble elastin fibers. (a) SDS-PAGE analysis of the binding ability of pseudolysin to insoluble elastin. BSA was used as a negative control. The bound and unbound fractions were analyzed by 15% SDS-PAGE. For protein staining, the gels were stained with 1.0% (wt/vol) Coomassie brilliant blue R-250. (Full-length gels are presented in Supplementary Fig. S1). (b) Effects of NaCl and nonionic detergents on the binding of pseudolysin to insoluble elastin. (Full-length gel is presented in Supplementary Fig. S2).

We first investigated whether pseudolysin can bind to elastin fibers. Because the optimum temperature for pseudolysin activity was shown to be around 60°C in previous study^29^, we performed our assays at 55°C in this study. After the activity of pseudolysin was inhibited with exogenous Zn^2+^, the elastin-binding ability of pseudolysin was assessed by SDS-PAGE. As shown in Fig. 1a, the amount of bound pseudolysin increased as the amount of elastin rose, indicating that pseudolysin can bind to elastin fiber at 55°C. We also tested the effect of temperature on the elastin-binding ability of pseudolysin The result showed that the elastin-binding ability of pseudolysin increased with temperature in the range of 4-55°C (Supplementary Fig. S4). It is also showed that pseudolysin still had significant elastin-binding ability at 37°C, the temperature of human body (Supplementary Figs. S4, S5).

To further explore the binding pattern, we examined the effects of nonionic detergents and NaCl on the elastin-binding ability of pseudolysin. As shown in Fig. 1b, three nonionic detergents, Tween 20, Tween 60 and Tween 80, all decreased the elastin-binding ability of pseudolysin, and NaCl at concentrations of 1.5 M and 2.0 M enhanced the binding, suggesting that hydrophobic interactions exist between pseudolysin and elastin. In addition, we also observed that lower concentrations of NaCl, namely 0.5 M and 1 M, reduced the binding of pseudolysin to insoluble elastin, which suggested that in addition to hydrophobic interactions, electrostatic interactions may exist between pseudolysin and elastin.

### Analysis of the cleavage sites of pseudolysin in bovine elastin

To identify the cleavage sites of pseudolysin in bovine elastin, the molecular masses of the peptides released from bovine elastin fibers were determined by LC-MS, and then the sequences of these peptides were determined based on the sequence of bovine tropoelastin. Finally, 108 cleavage sites of pseudolysin in bovine elastin were determined based on 75 released peptides (see Supplementary Table S1). Among these cleavage sites, Gly showed the highest occurrence in each site from P4 through to P4' likely because Gly is the most abundant residue in elastin. In addition, the P1’ positions of 70 sites were occupied by hydrophobic amino acids, Ala (13), Val (26), Leu (10), Ile (3), Pro (10), and Phe (8), whereas only 5 sites were occupied by hydrophilic amino acids, Lys (2), Thr (1), Ser (1), and Gln (1) (Table 1). The P1 position of these cleavage sites were also mostly occupied by hydrophobic amino acids, Ala (14), Val (16), Leu (8), Ile (3), Pro (13), and Phe (2), and only 7 sites were occupied by hydrophilic amino acids, Lys (4), Thr (1), and Arg (2). Moreover, Pro showed a high hit rate at sites from P3 through to P4' likely because this hydrophobic amino acid is the second most abundant residue in the hydrophobic domains of elastin. Altogether, these results indicate that pseudolysin prefers to attack peptide bonds with hydrophobic amino acid residues at the P1 and P1’ positions.

**Figure 2:**
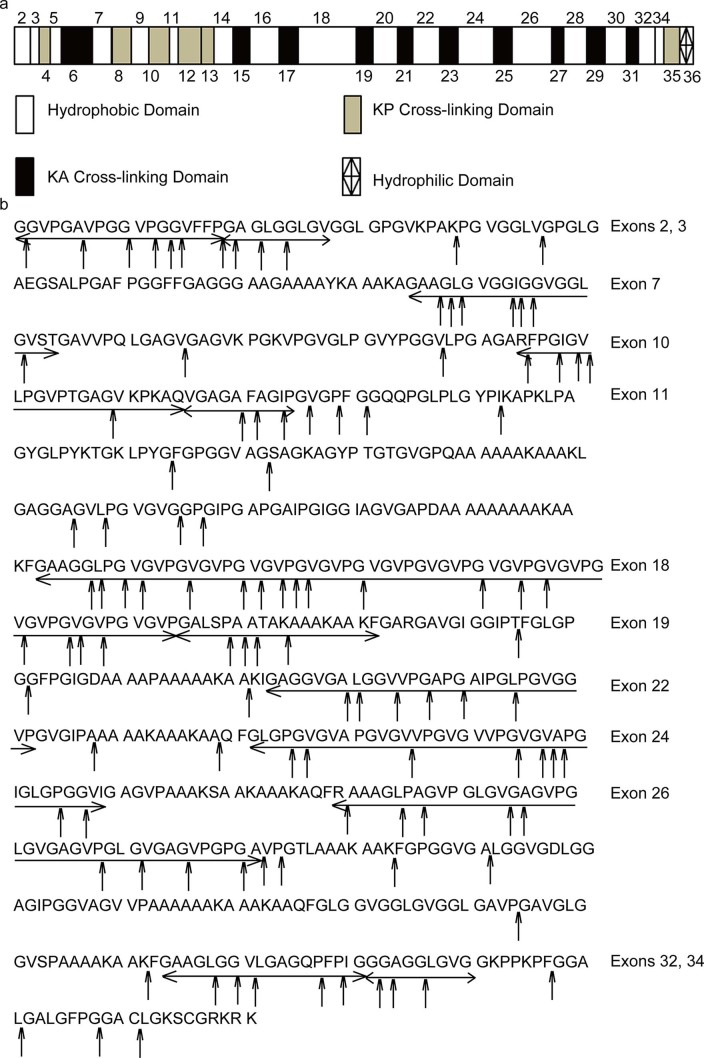
Analysis of the cleavage sites of pseudolysin in bovine elastin. (a) Domain map of bovine elastin. Hydrophilic cross-linking domains are further divided into domains with lysine pairs separated by one or more proline residues (KP cross-linking domains) and domains with lysine pairs separated by alanine residues (KA cross-linking domains). Exon 36 encodes a hydrophilic C-terminus assigned differently^22^. (b) Cleavage sites of pseudolysin on bovine elastin (SwissProt accession number P04985-1). Exons are underlined by an arrow. Cleavage sites were deduced from the sequences of the peptides released by pseudolysin from bovine elastin as shown in Supplementary Table S1 online.

Bovine tropoelastin is encoded by 36 exons. While exons 2, 3, 5, 7, 9, 11, 14, 16, 18, 20, 22, 24, 26, 28, 30, 32 and 34 encode hydrophobic domains, the others encode hydrophilic domains (Fig. 2a). The determined cleavage sites of pseudolysin on the tropoelastin sequence are marked by arrows in Fig. 2b. Most cleavage sites are on the domains encoded by exons 2, 3, 7, 10, 11, 18, 19, 22, 24, 26, 32 and 34. Except for 10 and 19, these domains are all hydrophobic domains. Thus, pseudolysin prefers to degrade the peptide bonds in the hydrophobic domains of elastin, suggesting that elastin degradation by pseudolysin may begin with the hydrophobic domains.

### *In situ* observation of bovine elastin hydrolysis by pseudolysin using light microscopy

**Figure 3:**
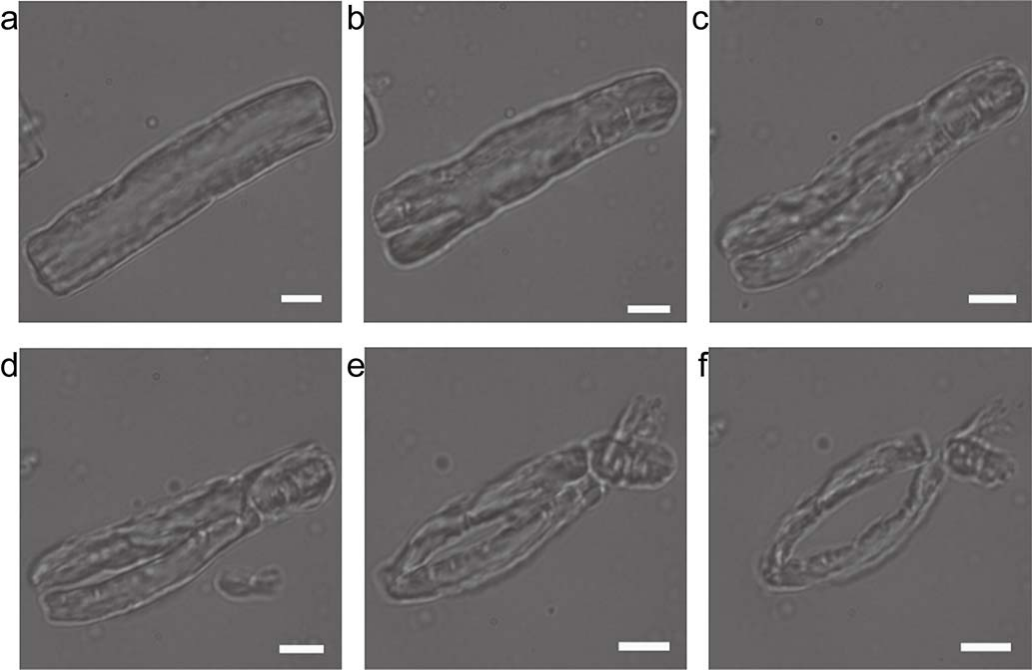
*In situ* observation of bovine elastin hydrolysis by pseudolysin using light microscopy. Trace amount of bovine elastin was added to a drop of 1 mg/ml pseudolysin in 50 mM Tris-HCl buffer (pH 8.0) on a glass slide. The sample was observed and photographed with an inverted microscope (Olympus IX71, Japan) at room temperature at 5 min intervals. Magnification is ×960. Scale bars: 5 μm.

The hydrolysis of bovine elastin fibers by pseudolysin was monitored *in situ* under an inverted microscopy. The degradation of a fiber of a suitable size (approximately 8 μm in width and 30 μm in length) and with an unbroken surface was examined (Fig. 3a). After 5 min of treatment with pseudolysin (1 mg/ml), multiple crevices appeared on the fiber surface, which were both longitudinal and transverse (Fig. 3b). The number and size of the crevices increased over time (Fig. 3c, d). The fiber was broken lengthwise by progressive longitudinal cracks and then was broken into pieces by progressive transverse cracks after 20~25 min of treatment (Fig. 3e, f).

### SEM observation of bovine elastin degradation by pseudolysin

**Figure 4:**
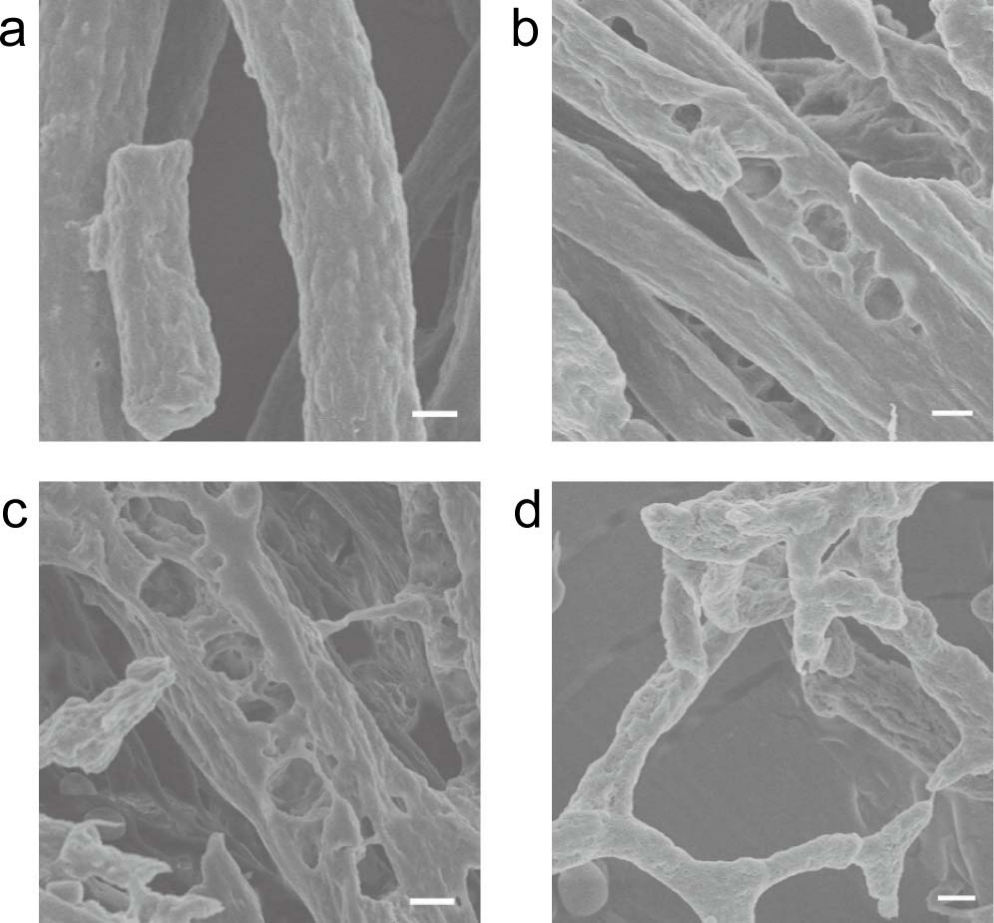
SEM observation of bovine elastin degradation by pseudolysin. Pseudolysin (0.05 mg/ml) in 50 mM Tris-HCl (pH 8.0) was incubated with 5 mg bovine elastin at 55°C with continuous stirring. The same reaction system without pseudolysin served as a control (a). After 2, 3, or 4 h, the reaction mixture was gently mixed with 200 μl deionized water, and the elastin fibers were then separated by a natural settling process on ice. This process was repeated twice for a complete washing of the elastin fibers. After dehydration by lyophilization, samples were observed under a Hitachi FE-S4800 SEM at 5.0 kV. Scale bars: 2 μm.

To examine the elastin degradation process in greater detail, the time-dependent progressive disintegration of elastin fibers treated with pseudolysin was further investigated by SEM. Fig. 4 shows the dramatic process by which the fiber was disrupted by pseudolysin under SEM. Untreated elastin fibers composed of tightly arranged fibrils showed diameters of 2-6 μm (Fig. 4a). After 2 h of treatment with 0.05 mg/ml pseudolysin, cavities appeared on the surface of the elastin fibers (Fig. 4b), which spread gradually in depth and width with increasing incubation time (Fig. 4c). After 4 h of treatment, intact elastin fibers disappeared, and there were only irregular pieces in the field of view (Fig. 4d).

### SEM observation of the degradation of cross-linked recombinant human tropoelastin spherules by pseudolysin

**Figure 5:**
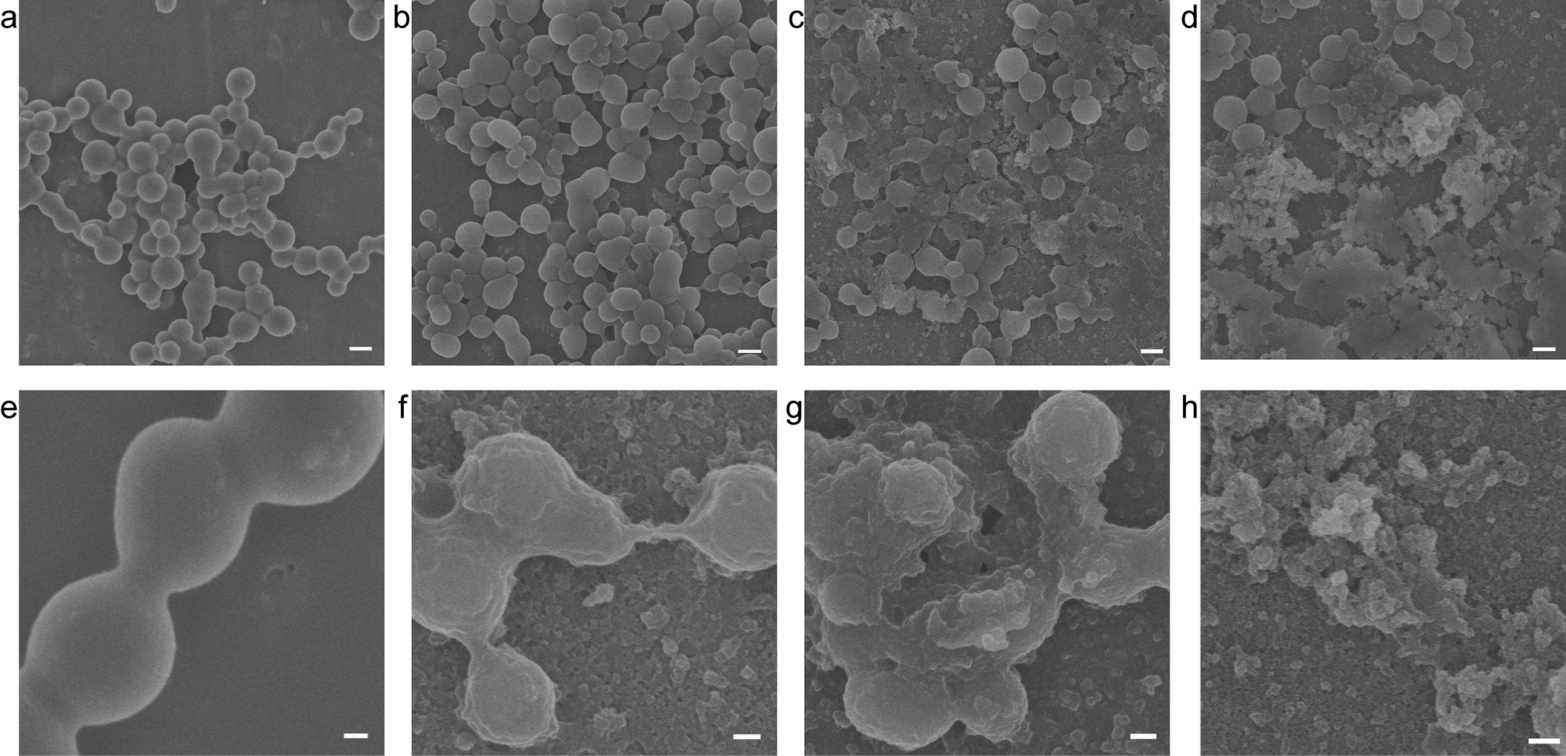
Degradation of cross-linked recombinant human tropoelastin clusters by pseudolysin. Tropoelastin (10 mg/ml) was cross-linked with 10 mM BS^3^ in 10 mM PBS for 30 min at 37°C (a and e). The cross-linked product was incubated with 0.1 mg/ml pseudolysin for 2 h (b and f), 3 h (c and g) and 4 h (d and h). e-h are the same samples as a-d, examined at a higher magnification as indicated by the scale bar. Scale bars in a-d: 1 μm. Scale bars in e-h: 200 nm.

To further confirm that pseudolysin preferred to degrade the peptide bonds in the hydrophobic domains of elastin, the degradation pattern of cross-linked recombinant human tropoelastin by pseudolysin was observed by SEM. Cross-linked human tropoelastin is a simple elastin analogue, in which the hydrophobic domains form spherules and the hydrophilic domains form cross-links between spherules. Under SEM, the spherules and cross-links in cross-linked tropoelastin were both clearly observed (Fig. 5a and e). After 1 h of treatment with 0.1 mg/ml pseudolysin, the spherules were still cross-linked (Fig. 5b) but appeared irregular (Fig. 5f), suggesting the decomposition of the spherules by pseudolysin. After 3 h of treatment, some of the spherules were completely degraded into floccules (Fig. 5c and g). The destroyed spherules began to release particles, indicating the loss of coacervation of these spherules due to the destruction of hydrophobic domains. After 4 h, most of the spherules were degraded into floccules (Fig. 5d and h). Throughout the entire degradation process, few single spherules were detectable, suggesting that cross-links were difficult to destroy by pseudolysin. Taken together, these results demonstrate that pseudolysin prefers to degrade hydrophobic domains during elastin degradation. The results also confirm that pseudolysin can destroy human elastin.

### Determination of the key residues in pseudolysin for elastin binding and catalysis

**Figure 6:**
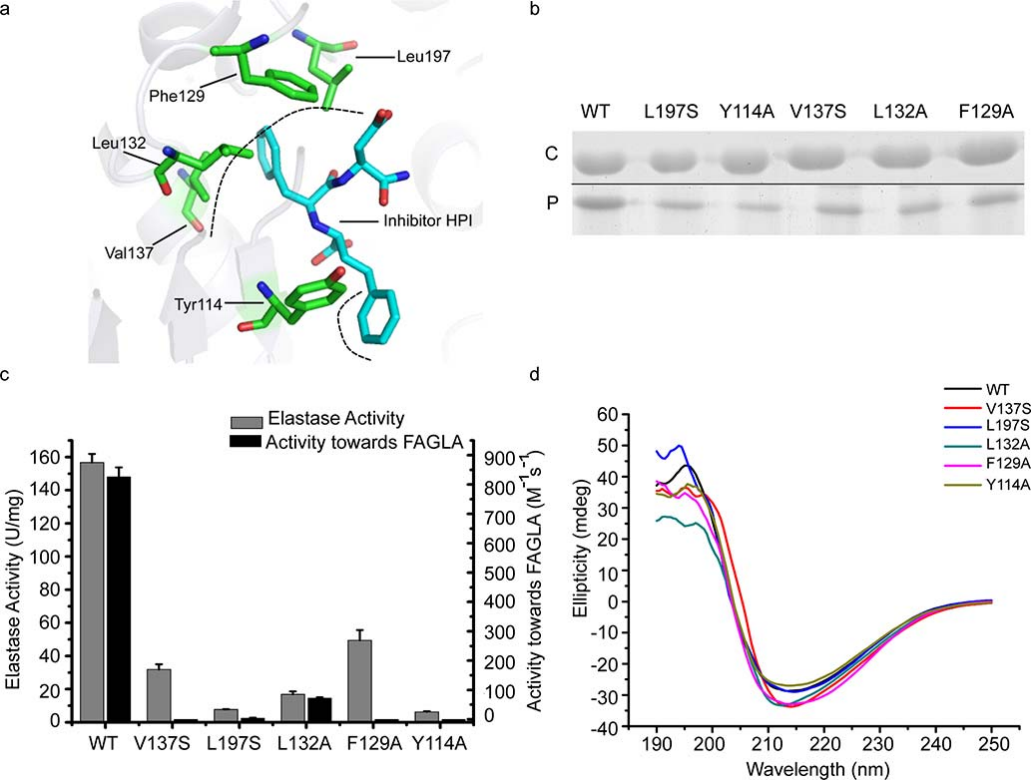
(a) Top view of the interaction of pseudolysin with the inhibitor HPI based on the structure of complexes between pseudolysin and HPI (PDB id: 1U4G). Black dashed lines represent the hydrophobic interactions. (b) SDS-PAGE analysis of the binding ability of pseudolysin and its mutants to insoluble elastin. ‘C’ represents the pseudolysin mixed with insoluble elastin in the experiment, and ‘P’ refers to the pseudolysin released from the precipitated insoluble elastin. (Full-length gel is presented in Supplementary Fig. S3). (c) Enzymatic activities of pseudolysin and its mutants towards elastin and FAGLA. The graph shows data obtained from triplicate experiments (mean ± S.D.). (d) CD spectra of pseudolysin and its mutants.

The crystal structure of pseudolysin has been determined^9^. To investigate the structural basis for the substrate specificity of pseudolysin toward elastin, the structure of the S1-S1’ sub-sites of pseudolysin were analyzed in detail. An analysis of the structure of pseudolysin complexed with the inhibitor HPI indicated that hydrophobic interactions are mainly formed between HPI and Val137, Leu197, Leu132, Phe129 and Tyr114 (Fig. 6a). To determine the importance of these hydrophobic amino acid residues in the S1-S1’ sub-sites for elastin binding, site-directed mutagenesis on these residues was introduced to decrease the residues’ hydrophobicity. Five site-directed mutations, V137S, L197S, L132A, F129A and Y114A, were constructed and expressed. The binding ability and enzymatic activity of these mutants toward elastin fibers were assayed and compared with that of wild-type pseudolysin. All of these mutations significantly reduced the elastin binding ability of pseudolysin (Fig. 6b) and also resulted in a considerable decrease in the enzymatic activity toward both elastin and the dipeptide substrate 3-(2-furylacryloyl)glycyl-L-leucine amide (hereafter referred to as FAGLA) (Fig. 6c). A comparison of the circular dichroism (CD) spectra of pseudolysin and its mutants indicated that these mutations caused only slight structural changes in pseudolysin (Fig. 6d). Therefore, the decrease in the elastin-binding ability and the enzymatic activity of the mutants were mainly due to amino acid replacement, rather than structural change. These results suggest that the hydrophobic residues in the S1-S1’ sub-sites, Val137, Leu197, Leu132, Phe129 and Tyr114, may form hydrophobic interactions with the hydrophobic residues in the hydrophobic domains of elastin, thereby playing key roles in the binding of pseudolysin to elastin during elastin degradation.

## Discussion

Pseudolysin is the major extracellular virulence factor of the pathogenic bacterium *P. aeruginosa*. It destroys human tissues by solubilizing elastin^4^. In recent years, the fine structure of elastin fibers has been better understood^21^, which provides the possibility to reveal the mechanism of action of pseudolysin on elastin fibers. In this study, we investigated the mechanism of action of pseudolysin on bovine elastin fibers by biochemical assay, microscopy and structural analysis. Bovine tropoelastin (SwissProt accession number P04985-1) shows a high identity of 75% with human tropoelastin (accession number P11502), and the domain structures of bovine and human tropoelastin are similar^22^,^23^. The organization of the elastin fiber network is also similar to that observed in bovine tail and human^24^,^25^. These previous findings support the use of bovine elastin fibers as a suitable material for studying the action of pseudolysin on human elastin.

Recent studies have shown that a tropoelastin molecule consists of an elastin N-terminal coil region and a cell-interactive C-terminal foot region, which are linked together by a highly exposed bridge region. A model for elastin assembly indicates that juxtaposed domains 19 and 25 on one tropoelastin molecule are cross-linked to domain 10 on an adjacent tropoelastin molecule to form a molecule of desmosine. These assembled tropoelastin monomers form covalently bonded molecules with a diameter of 5 nm, which are further cross-linked to form more complex structures. It is also reported that the lateral association between bonded molecules is driven by hydrophobic interactions^26^. Based on these studies, it can be concluded that the surfaces of the filaments in a cross-linked elastin fiber are covered with hydrophobic domains and hydrophilic domains are mainly distributed among the filaments for cross-linking.

Microscopy has been confirmed to be a useful method for investigating the action of proteases on macroscopic insoluble protein fibers, such as collagen and elastin^27^,^28^. SEM combined with biochemistry assay showed that the M23 protease pseudoalterin from a marine bacterium first released elastin filaments from elastin fiber by hydrolyzing cross-links in the hydrophilic domains during elastin degradation^28^. Cleavage site analysis indicates that this M23 protease preferred to cleave the glycyl bonds in hydrophobic regions and the peptide bonds Ala-Ala, Ala-Lys, and Lys-Ala involved in cross-linking^28^. LM and SEM showed that, unlike the behavior observed for pseudoalterin, the degradation of elastin fiber by pseudolysin began at certain spots on the surface of elastin fibers, forming enlarged crevices and cavities. This result, combined with the results obtained from cleavage site analysis, indicates that elastin degradation by pseudolysin began with the hydrophobic domains on the surface of elastin fibers, followed by the progressive degradation of macroscopic elastin fibers into primary structural elements. SEM observation of the degradation of cross-linked human tropoelastin by pseudolysin also confirmed that pseudolysin preferred to attack the spherules coacervated by hydrophobic domains rather than the cross-links.

Based on our results, the mode of action of pseudolysin on elastin degradation can be elucidated. When pseudolysin molecules meet with an elastin fiber, they bind to the hydrophobic domains on the surface of the elastin fiber. After binding, the molecules begin to attack the sensitive bonds with hydrophobic amino acid residues at the P1 and P1’ positions, resulting in the appearance of crevices and cavities on the elastin fiber. The crevices and cavities extend on the surface of the elastin fiber over time until the fiber is broken into pieces, which are further degraded into small peptides and free amino acids.

Our analysis of the cleavage sites of pseudolysin on elastin showed that pseudolysin prefers hydrophobic residues at the P1’ and P1 positions, which is consistent with a previous report indicating that pseudolysin favors hydrophobic and aromatic amino acid residues at the P1’ position based on its effect on synthetic peptides^6^. Although the structure of pseudolysin has been determined, the mechanism by which pseudolysin recognizes elastin remains unclear. Because there has been no reported structure of pseudolysin complexed with a substrate to date, we used the structure of pseudolysin complexed with the inhibitor HPI as a reference to predict the possible key residues responsible for elastin binding. Based on the structure of this complex, the S1’ sub-site of pseudolysin was determined to be mainly composed of Val137, Leu197, Leu132, Phe129, His140, Ile186 and Gly187, and the S1 sub-site of pseudolysin was determined to be composed of Tyr114, Trp115, Tyr157 and Asn112. Val137, Leu197, Leu132, and Phe129 in the S1’ sub-site and Tyr114 in the S1 sub-site are the key residues involved in hydrophobic interactions with HPI. Therefore, we performed site-directed mutations on these hydrophobic residues to investigate their importance in the binding of pseudolysin to elastin. Our results indicate that the hydrophobic residues in the S1’ sub-site, Val137, Leu197, Leu132, and Phe129, and Tyr114 in the S1 sub-site played key roles in the binding of pseudolysin to insoluble elastin. These aliphatic and aromatic residues in the S1-S1’ sub-sites of pseudolysin may serve as the targets for designing specific drugs for blocking the elastin binding of pseudolysin in *P. aeruginosa* infection*.*

In summary, based on biochemistry assay, microscopy and site-directed mutation, we revealed the elastinolytic mechanism of pseudolysin and some key residues for elastin binding. Our results provide a better understanding of the pathogenesis of *P. aeruginosa.*

## Methods

Recombinant human tropoelastin was kindly provided by Anthony S. Weiss from the University of Sydney, Australia. Elastin-orcein, the chemical cross-linker bis(sulfosuccinimidyl) suberate (BS^3^), bovine serum albumin (BSA), and bovine neck ligament elastin isoform 1 (SwissProt accession number P04985–1, hereafter referred to as bovine elastin) were obtained from Sigma (USA). FAGLA was synthesized by ChinaPeptides Co., Ltd. (China). Recombinant pseudolysin was expressed in *Escherichia coli* and purified as previously described^29^.

### Assay of the binding ability of pseudolysin to insoluble elastin

The binding ability of pseudolysin to insoluble elastin was analyzed using the method of Valenzuela *et al.* with minor modifications^30^. Pseudolysin (0.2 mg/ml) was dissolved in 50 mM Tris-HCl (pH 8.0) containing 10 mM Zn^2+^ (hereafter referred to as buffer A) to inhibit its elastinolytic activity. Then, 0.2 ml enzyme solution (40 micrograms pseudolysin) was mixed with 5, 3, 1 or 0.5 mg elastin and incubated at 55°C for 40 min with stirring. Subsequently, samples were centrifuged for 10 min at 13,000 rpm and 4°C. The supernatants were removed for SDS-PAGE analysis; the pellets were washed twice with buffer A, then resuspended with an equivalent volume of loading buffer and boiled for 10 min to release the bound protein from elastin. The protein in the supernatant and the protein released from the precipitated elastin were analyzed by 15% SDS-PAGE. For protein staining, the gels were stained with 1.0% (w/v) Coomassie brilliant blue R-250. BSA was used as a negative control in place of pseudolysin in the reaction mixture.

### Assay of the effect of temperature on the binding ability of pseudolysin to insoluble elastin

Pseudolysin (0.1 mg/ml) was dissolved in 50 mM buffer A to inhibit its elastinolytic activity. Then, 0.2 ml enzyme solution was mixed with 3 mg elastin and incubated at 4, 25, 37 or 55°C for 40 min with stirring. Subsequently, samples were centrifuged for 10 min at 13,000 rpm and 4°C. The pellets were washed twice with buffer A, then resuspended with an equivalent volume of loading buffer, and boiled for 10 min to release the bound protein from elastin. The bound fractions were analyzed by 15% SDS-PAGE followed by Coomassie blue staining.

### Effects of NaCl and nonionic detergents on the binding of pseudolysin to insoluble elastin

This experiment was performed using the method of Itoi *et al.*^31^ Pseudolysin (0.2 mg/ml, 0.2 ml) inactivated in buffer A was mixed with 10 mg elastin and incubated at 55°C for 60 min with stirring. After incubation, the mixture was centrifuged for 10 min at 13,000 rpm and 4°C. The precipitate was washed twice in buffer A and resuspended in 80 μl buffer A containing nonionic detergent (1% Tween 20, 1% Tween 60, or 1% Tween80, v/v) or NaCl (2.0 M, 1.0 M, 1.5 M or 0.5 M). The precipitate resuspended in 80 μl buffer A served as a control. The mixtures were incubated for 120 min at 55°C and then centrifuged. Precipitates were treated as described above and analyzed by 15% SDS-PAGE. For protein staining, SDS-PAGE gels were stained with 1.0% (w/v) Coomassie brilliant blue R-250.

### Protein determination and enzyme assays

Protein concentrations were determined by using a BCA protein assay kit (Thermo, USA) according to the manufacturer’s instructions. The elastinolytic activity of pseudolysin was measured as previously described^32^. Briefly, the enzyme was incubated at 60°C with 5 mg elastin-orcein in 0.25 ml 50 mM Tris-HCl (pH 7.5) with continuous stirring for 1 h, and then the residual elastin-orcein was removed by centrifugation. The absorption of the supernatant at 590 nm was measured. One unit of enzyme activity was defined as the amount of enzyme that caused an increase in 0.01 unit of absorbance at 590 nm per min. The enzymatic activity of pseudolysin toward FAGLA was assessed using Feder's method^29^.

### Cleavage sites for pseudolysin on bovine elastin

Bovine elastin (30 mg) was incubated at 55°C with 0.2 mg pseudolysin in 0.5 ml 50 mM Tris-HCl (pH 8.0) with continuous stirring. Bovine elastin in the same buffer without pseudolysin served as a negative control. After the mixture had no visible insoluble elastin, the samples were boiled for 10 min to terminate the reaction, and the supernatant was loaded onto an LC-MS (micro TOF-QII LC-MS/MS, Bruker) after high-speed centrifugation to determine the molecular mass of the released peptides. The sequences of the released peptides were analyzed by MASCOT MS/MS Ion Research and Expasy instruments (www.expasy.org).

### *In situ* observation of bovine elastin hydrolysis by pseudolysin using light microscopy

A drop of pseudolysin (1 mg/ml in 50 mM Tris-HCl, pH 8) was placed on a glass slide with a trace amount of bovine elastin powder. The drop was slowly coated with a coverslip from one side to avoid bubble formation. The prepared sample was moved to the objective table of an inverted microscopy (Olympus IX71, Japan) for observation and photographed at 5 min intervals at room temperature.

### Observation of bovine elastin degradation by pseudolysin under scanning electron microscopy

Five milligrams of bovine elastin fibers was incubated with 0.2 ml pseudolysin (0.05 mg/ml) in 50 mM Tris-HCl (pH 8.0) at 55°C with continuous stirring. Bovine elastin fibers in the buffer without pseudolysin served as a control. At 2, 3 or 4 h of digestion, the reaction mixture was gently mixed with 200 μl deionized water, and then elastin fibers were separated by a natural settling process on ice. This process was repeated twice to completely wash the elastin fibers. After dehydration by lyophilization, samples were mounted on a metal stub and coated with a 5 nm layer of platinum before examination with a Hitachi FE-S4800 SEM at 5.0 kV.

### Preparation, degradation, and observation of cross-linked recombinant human tropoelastin

Cross-linked recombinant human tropoelastin isoform SHELdelta26A was prepared as previously described^28^. Briefly, 1 μl cold chemical cross-linker BS^3^ (10 mM) was added to 9 μl tropoelastin (10 mg/ml), which was dissolved in 10 mM phosphate-buffered saline (PBS) (pH 7.4). The mixture was spread thinly on aluminum foils and incubated at 37°C for 30 min for cross-linking. The cross-linked tropoelastin was washed with PBS repeatedly, followed by hydrolysis with 0.1 mg/ml pseudolysin in Tris-HCl buffer (pH 8.0) for 2, 3 or 4 h at 55°C. The residual cross-linked layer was washed and dehydrated^27^. The samples on aluminum foils were sputter-coated and examined with a Hitachi FE-S4800 SEM at 5.0 kV.

### Site-directed mutagenesis of pseudolysin

Site-directed mutations were introduced by overlapping extension-PCR with a vector containing the pseudolysin gene as a template, which was constructed as previously described^29^. Mutations were introduced by the primers with a single mutation. The mutated genes were subcloned into pET-22b(+) and transformed into *E. coli* BL21(DE3). All mutations were confirmed by enzyme digestion and nucleotide sequencing. All mutants were expressed and purified as wild-type pseudolysin^29^. The binding ability and enzymatic activity of the mutants were assayed as wild-type pseudolysin. The CD spectra of purified pseudolysin and all of the mutants were assayed on a Jasco J810 spectropolarimeter (Japan) with a bandwidth of 2 nm, a response time of 1 s, and a scan speed of 500 nm/min. Each spectrum was the average of three scans monitored between 190 and 250 nm. The path length of the cuvette was 0.1 cm. The spectra were reported in terms of molar ellipticity.

## Author Contributions

X.L.C. and Y.Z.Z. designed the research; J.Y. and H.L.Z., performed the research and wrote the paper; L.Y.R. And C.Y.L. contributed to structural analysis and mutational study; X.Y.Z., H.N.S., M.S. and B.C.Z. analyzed data. All authors discussed the results and participated in manuscript revision.

## Supplementary Material

Supplementary Information Supplementary Information

## Figures and Tables

**Table 1:** Specificity matrix of pseudolysin to bovine elastin fibers based on the determined 108 cleavage sites.

Amino acid	P4		P3	P2	P1	P1’	P2’	P3’	P4’
G	47		35	41	44	33	46	39	41
A	17		14	18	14	13	17	8	13
V	16		20	10	16	26	13	28	14
L	8		7	4	8	10	4	5	10
I	2		5	2	3	3	4	3	5
P	8		15	26	13	10	16	17	14
K	3		3		4	2	1	2	5
R	1				2				
F	2		4	4	2	8	3	2	3
T	1		1		1	1	2	2	1
C					1			1	
S	2		1	1		1	1		1
Y			1	1					
Q	1		2	1		1	1	1	1
