# Supplementary material for: Mechanistic Insights into Elastin Degradation by Pseudolysin, the Major Virulence Factor of the Opportunistic Pathogen Pseudomonas aeruginosa
Source: Sci Rep. 2015 Apr 23;5:9936. doi: 10.1038/srep09936 (PMC4407726; doi:10.1038/srep09936)
Supplement: Supplementary Information [file srep09936-s1.pdf]

Mechanistic Insights into Elastin Degradation by Pseudolysin, the Major Virulence Factor of the Opportunistic Pathogen *Pseudomonas aeruginosa*

Supplementary Data

Jie Yang[1,2], Hui-Lin Zhao[1,2,#], Li-Yuan Ran[1,2], Chun-Yang Li[1,2], Xi-Ying Zhang[1,2], Hai-Nan Su[1,2], Mei Shi[1,2], Bai-Cheng Zhou[2], Xiu-Lan Chen[1,2,3,*], Yu-Zhong Zhang[1,2,3]

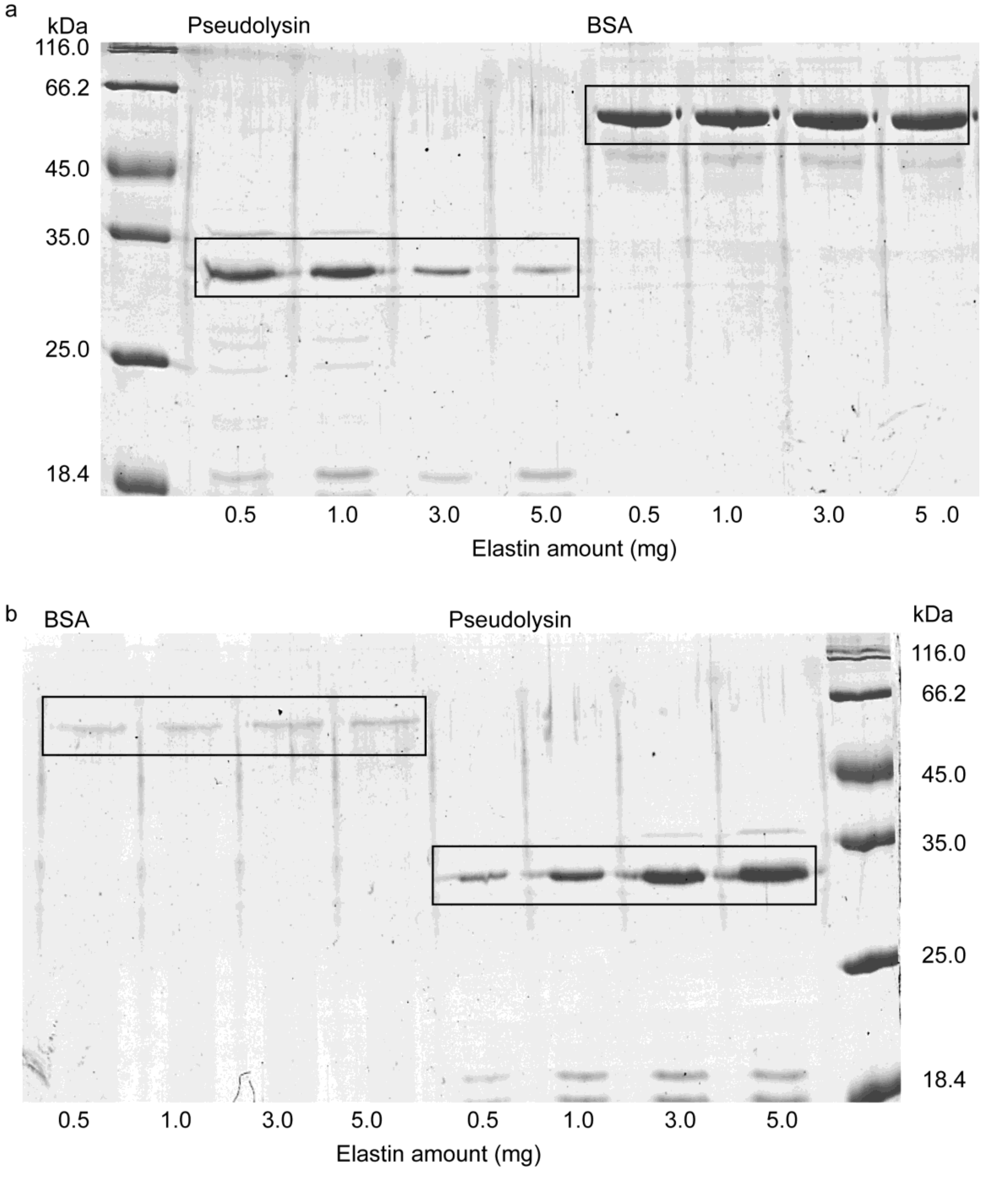

Supplementary Figure S1 | SDS-PAGE analysis of the binding ability of pseudolysin to insoluble elastin. BSA was used as a negative control. The unbound (a) and bound (b) fractions were analyzyed by 15% SDS-PAGE. For protein staining, the gels were stained with 1.0% (wt/vol) Coomassie brilliant blue R-250. (Full-length gels of Figure 1a).

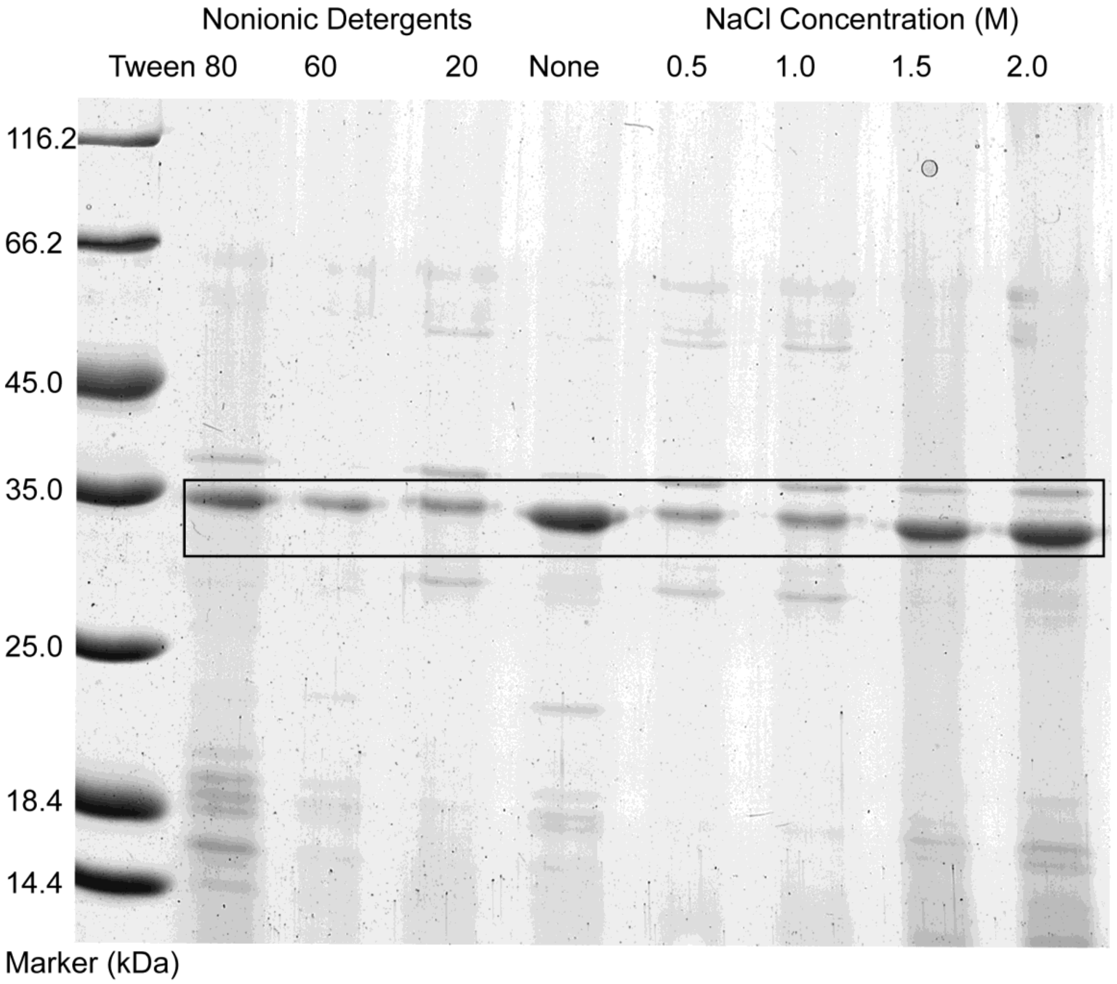

Supplementary Figure S2 | Effects of nonionic detergents and NaCl on the binding of pseudolysin to insoluble elastin. The samples were analyzyed by 15% SDS-PAGE. For protein staining, the gels were stained with 1.0% (wt/vol) Coomassie brilliant blue R-250. (Full-length gel of Figure 1b).

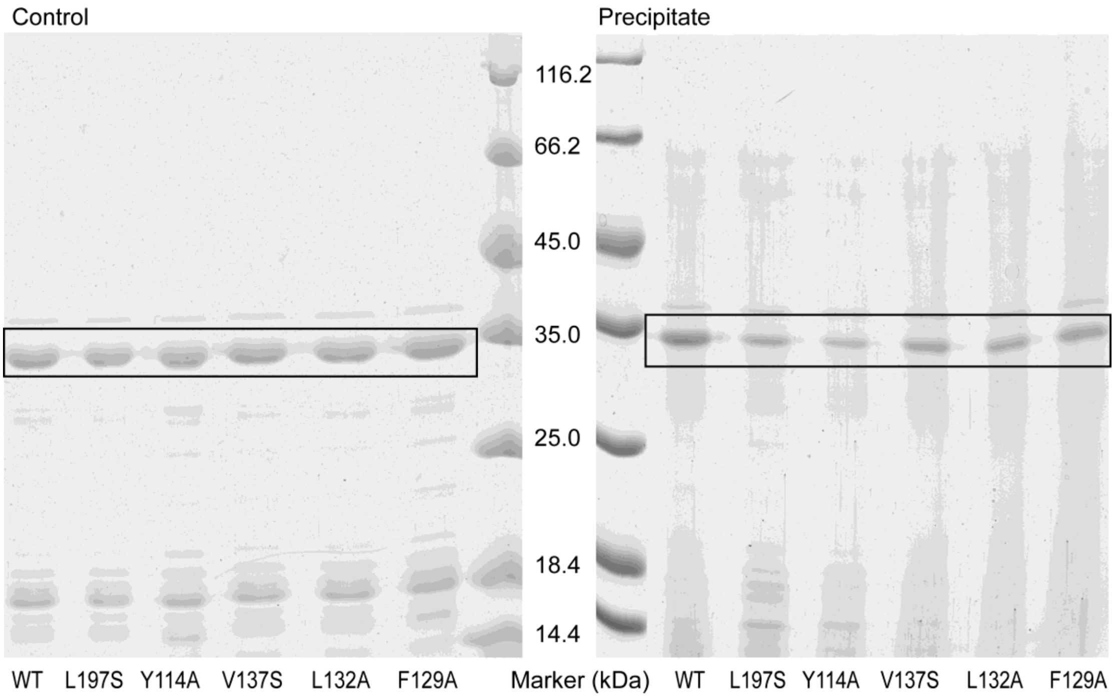

Supplementary Figure S3 | SDS-PAGE analysis of the binding ability of pseudolysin and its mutants to insoluble elastin. The samples were analyzed by 15% SDS-PAGE. For protein staining, the gels were stained with 1.0% (wt/vol) Coomassie brilliant blue R-250. 'Control' represents the proteins mixed with insoluble elastin in the experiment, and 'Precipitate' refers to the pseudolysin released from the precipitated elastin. (Full-length gel of Figure 6b).

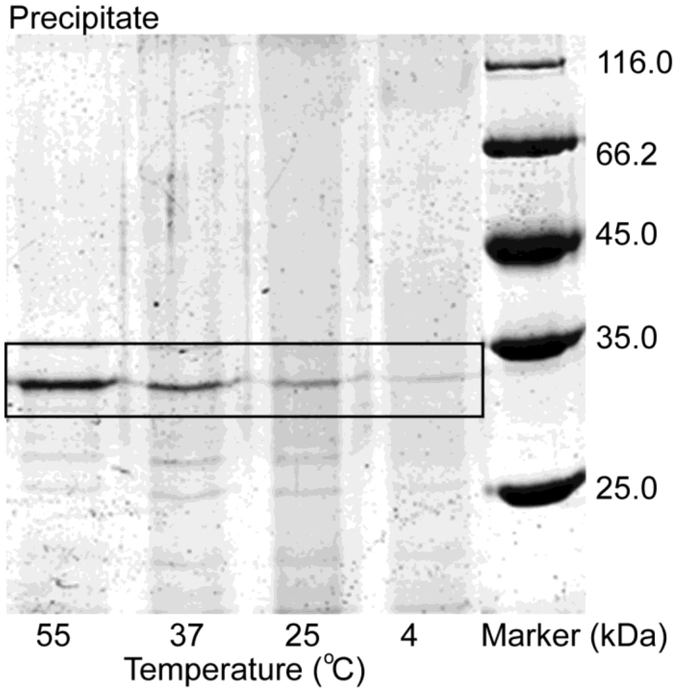

Supplementary Figure S4 | SDS-PAGE analysis of the binding ability of pseudolysin to insoluble elastin under different temperatures. The bound fractions were analyzed by 15% SDS-PAGE followed by Coomassie blue staining. ‘Precipitate’ refers to the pseudolysin released from the precipitated elastin.

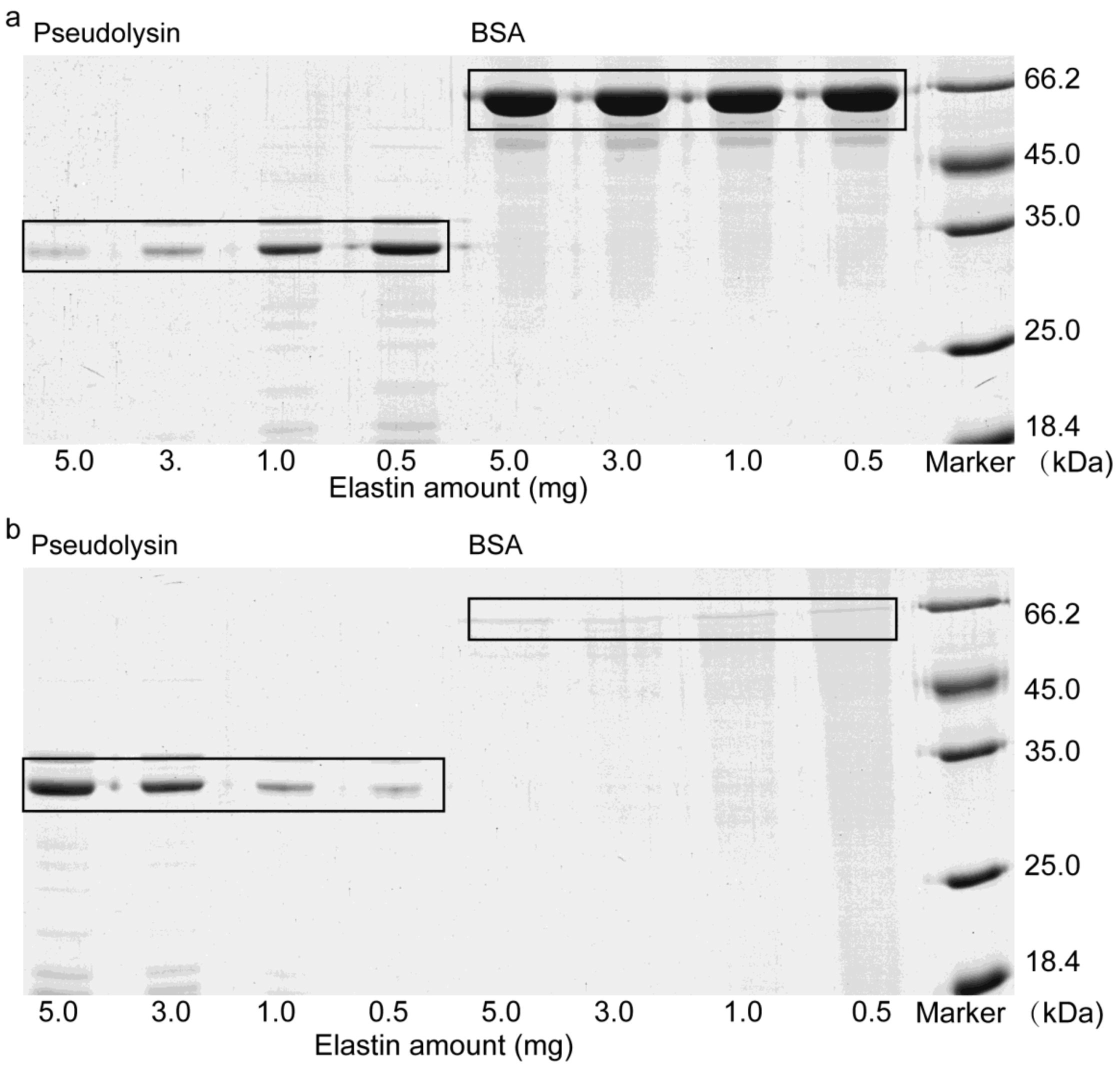

Supplementary Figure S5 | SDS-PAGE analysis of the binding ability of pseudolysin to insoluble elastin at 37 ℃. BSA was used as a negative control. The unbound (a) and bound (b) fractions were analyzyed by 15% SDS-PAGE. For protein staining, the gels were stained with 1.0% (wt/vol) Coomassie brilliant blue R-250.

Supplementary Table S1 | The determined molecular masses and sequences of the 75 peptides released from bovine elastin fibers by pseudolysin.

Number	MH^+ Observed (Da)	Mr (expt) (Da)	Peptide[a]	Position
1	710.38	709.38	(G)GVPGAVPGG(V)	28 - 36
2	639.34	638.34	(A)VPGGVPGG(V)	33 - 40
3	1129.58	1128.57	(A)VPGGVPGGVFFP(G)	33 - 44

4	657.84	1313.66	(A)VPGGVPGGVFFPGAG(L)	33 - 47
5	819.45	818.45	(G)VPGGVFFP(G)	37 - 44
6	623.34	622.33	(P)GGVFFP(G)	39 - 44
7	623.33	622.33	(G)GVFFPG(A)	40 - 45
8	751.39	750.39	(G)GVFFPGAG(L)	40 - 47
9	566.31	565.31	(G)VFFPG(A)	41 - 45
10	601.33	600.32	(P)GAGLGGLG(V)	45 - 52
11	598.31	597.31	(K)PGVGGLV(G)	65 - 71
12	629.36	628.35	(A)GLGVGGIG(G)	114 - 121
13	572.33	571.33	(G)LGVGGIG(G)	115 - 121
14	402.22	401.21	(L)GVGGI(G)	116 - 120
15	629.36	628.35	(G)IGGVGGLG(V)	120 - 127
16	622.34	1864.00	(G)IGGVGGLGVSTGAVVPQLGAGV(G)	120 - 141
17	471.76	941.51	(V)LPGAGARFPG(I)	164 - 173
18	556.81	1111.60	(V)LPGAGARFPGIG(V)	164 - 175
19	490.25	489.25	(R)FPGIG(V)	171 - 175
20	867.48	866.47	(G)VLPGVPTGAG(V)	176 - 185
21	768.43	767.42	(V)LPGVPTGAG(V)	177 - 185
22	561.29	560.28	(A)FAGIPG(V)	197 - 202
23	871.46	870.45	(F)AGIPGVGPFG(G)	198 - 207
24	426.23	425.22	(I)PGVGP(F)	201 - 205
25	476.24	475.24	(G)VGPFG(G)	203 - 207
26	566.30	1695.89	(G)VGPFGGQQPGLPLGYPI(K)	203 - 219
27	661.33	660.32	(G)FGPGGVAG(S)	241 - 248
28	754.44	753.44	(A)GVLPGVGVG(G)	282 - 290
29	639.37	638.37	(L)PGVGVGGP(G)	285 - 292
30	695.40	694.39	(G)LPGVGVPG(V)	334 - 341
31	552.81	1103.61	(G)LPGVGVPGVGVPG(V)	334 - 346
32	582.32	581.31	(L)PGVGVPG(V)	335 - 341
33	545.82	1089.63	(L)PGVGVPGVGVPGV(G)	335 - 347
34	837.49	836.48	(G)VGVPGVGVPG(V)	337 - 346
35	546.82	1091.62	(G)VGVPGVGVPGVGV(P)	337 - 349
36	623.85	1245.69	(G)VGVPGVGVPGVGVPG(V)	337 - 351
37	828.47	1654.92	(G)VGVPGVGVPGVGVPGVGVPG(V)	337 - 356
38	825.47	2473.38	(G)VGVPGVGVPGVGVPGVGVPGVGVPGVGVPG(V)	337 - 366
39	1234.70	3701.08	(G)VGVPGVGVPGVGVPGVGVPGVGVPGVGVPGVGVPGVGVPGVGVPG(V)	337 - 381
40	738.40	737.39	(V)GVPGVGVPG(V)	338 - 346
41	545.82	1089.62	(V)GVPGVGVPGVGVP(G)	338 - 350
42	1147.62	1146.61	(V)GVPGVGVPGVGVPG(V)	338 - 351
43	835.50	2503.47	(G)VGVPGVGVPGVGVPGVGVPGVGVPGALSPA(A)	367 - 396
44	1089.57	2177.13	(V)PGVGVPGVGVPGVGVPGVGVPGALSP(A)	370 - 395

45	1233.69	2465.36	(G)VGVPGVGVPGVGVPGVGVPGALSPAATAK(A)	372 - 400
46	829.44	1656.86	(V)GVPGVGVPGVGVPGALSPAA(T)	378 - 397
47	1106.60	1105.59	(V)GVPGVGVPGALSP(A)	383 - 395
48	511.31	1020.61	(V)PGVGVPGALSPA(A)	385 - 396
49	547.28	546.27	(T)FGLGPG(G)	422 - 427
50	729.38	728.37	(A)KIGAGGVGA(L)	448 - 456
51	485.24	484.23	(L)GGVVPG(A)	458 - 463
52	497.25	496.25	(V)VPGAPG(A)	461 - 466
53	538.81	1075.60	(L)PGVGGVPGVGIPA(A)	472 - 484
54	618.31	617.31	(A)QFGLGP(G)	496 - 501
55	754.45	753.44	(G)VGVAPGVGV(V)	503 - 511
56	780.41	779.41	(V)VPGVGVVPG(V)	512 - 520
57	780.45	779.44	(G)VAPGIGLGP(G)	523 - 531
58	795.43	794.42	(V)APGIGLGPGG(V)	524 - 533
59	610.34	609.34	(A)PGIGLGP(G)	525 - 531
60	402.31	401.30	(R)AAAGL(P)	557 - 561
61	655.37	654.36	(A)GVPGLGVG(A)	564 - 571
62	1013.58	2025.14	(V)GAGVPGLGVGAGVPGLGVGAGVPGPG(A)	571 - 596
63	1008.63	1007.62	(P)GLGVGAGVPGPGA(V)	585 - 597
64	402.22	1203.64	(P)GLGVGAGVPGPGAVP(G)	585 - 599
65	710.41	709.40	(G)VGAGVPGPG(A)	588 - 596
66	426.23	425.22	(G)VPGPG(A)	592 - 596
67	661.33	660.32	(K)FGPGGVGA(L)	610 - 617
68	1006.55	2011.08	(P)GAVGLGGVSPAAAAKAAKFGAAGL(G)	671 - 694
69	649.32	648.31	(K)FGAAGLGG(V)	689 - 696
70	677.86	1353.70	(G)VLGAGQPFPIGGGAGG(L)	697 - 712
71	786.40	785.40	(V)LGAGQPFP(I)	698 - 705
72	490.26	489.25	(P)FPIGG(G)	704 - 708
73	547.31	546.30	(P)FPIGGG(A)	704 - 709
74	402.21	1203.62	(F)GGALGALGFPGGAC(L)	724 - 737
75	618.32	617.31	(L)GALGFPG(G)	728 - 734

[a] The sequence of each peptide was determined by liquid chromatography-mass spectrometry and MASCOT MS/MS Ion Research tools. The left bracketed residue of each peptide indicates the P1-site residue of the left cleavage site. The right bracketed residue of each peptide indicates the P1'-site residue of the right cleavage site.
